# A decision model on the repair and maintenance of shipping containers

**DOI:** 10.1186/s41072-020-00070-2

**Published:** 2020-10-23

**Authors:** Niclas Hoffmann, Robert Stahlbock, Stefan Voß

**Affiliations:** 1 University of Innsbruck, Institute of Mechatronic, Technikerstraße 13, Innsbruck, 6020 Austria; 2 University of Hamburg, Institute of Information Systems (IWI), Von-Melle-Park 5, Hamburg, 20146 Germany; 3 FOM Hochschule für Oekonomie und Management, Essen/Hamburg, Germany

**Keywords:** Container logistics, Decision support, Repair and maintenance

## Abstract

The use of shipping containers for the transport of goods has become indispensable and a crucial factor for globalization by providing inexpensive and safe transport opportunities. It is expected that the number of globally operating containers will increase in the near future. Despite a high technical modernisation of the logistic chain, the container still faces a risk of damage at any time and any place within the transport chain. In principle, a container is taken out of service, when a damage is recognized. Different causes of damage exist and various types of damage could occur to the container, ranging from minor to substantial major ones that do not permit the continued proper use of the container. Thus, an individual decision on repair and maintenance (R&M) for each damaged container is necessary. Aside from technical aspects, it has to be decided from an economical perspective whether a repair should be performed. A profound decision should consider various criteria like, e.g., repair costs, lifespan of the container, future yields and possible sales price. Based on a regulatory, practical, and scientific view, this paper proposes a multi-criteria decision model for the economic decision on the R&M of a damaged container. Implemented in Microsoft Excel, this decision model is easily applicable. The user can deduce a first (limited) guidance for dealing with a respective damaged container based on its current state and general market conditions.

## Introduction to shipping containers

**Fig. 1:**
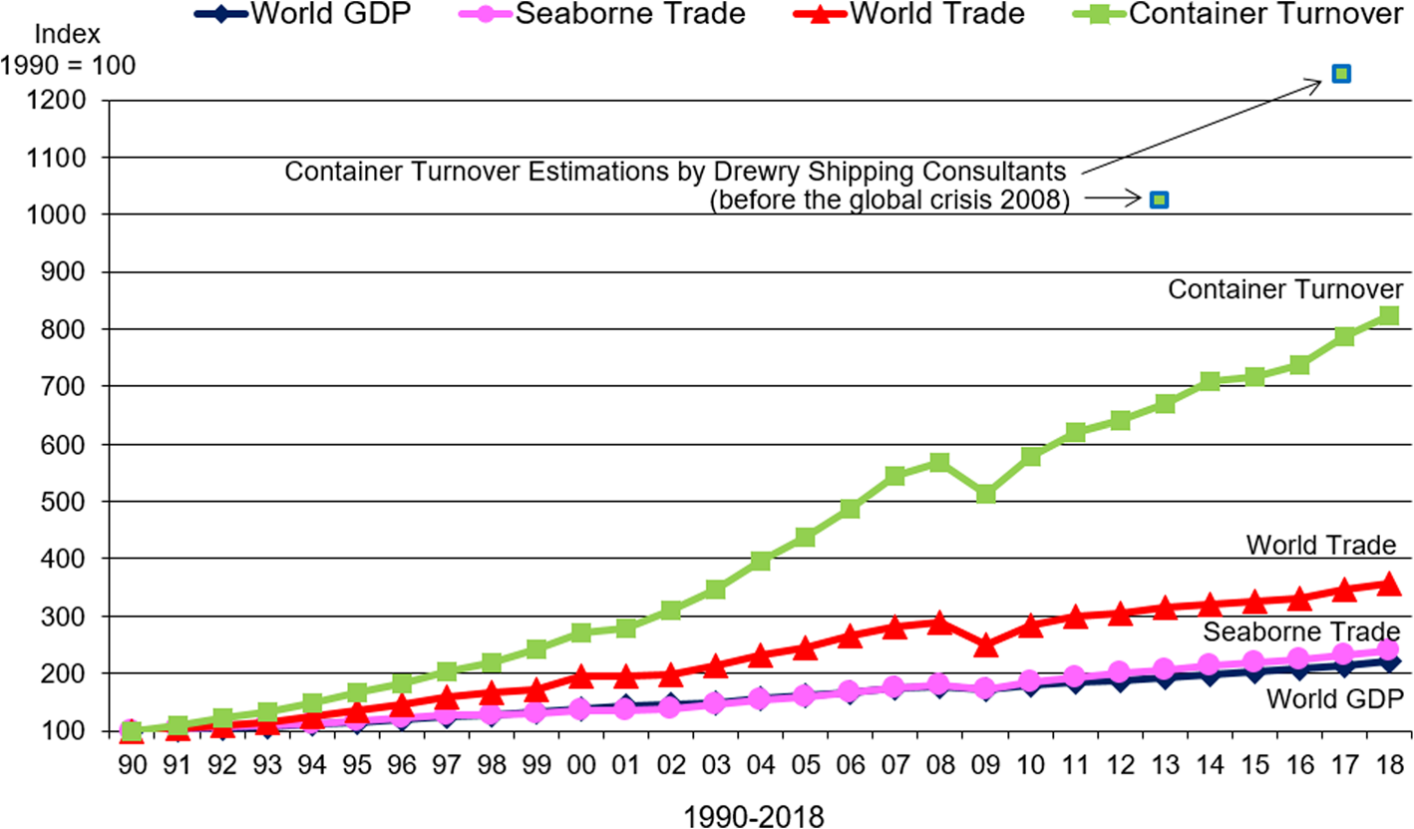
Container turnover, world trade, seaborne trade, and world GDP 1990–2018 with a forecast by Drewry Shipping Consultants for the container turnover for 2012 to 2016 before the global crisis 2008/2009; Data sources: United Nations Conference on Trade and Development (UNCTAD)–Secretariat (2019) and previous editions

The shipping container was invented by Malcolm McLean in North America in 1956. Its launch in the ocean shipping market led to a drastic reduction of the vessel turnaround time by enabling and establishing much more efficient (un)loading procedures (United Nations – Economic and Social Council – CEPAL – Economic Commission for Latin America 1982; Levinson 2016). Due to standardization conducted by the ‘International Organization for Standardization’ (ISO) since 1961, the logistic chain could be permanently improved and accelerated. Standard sizes of containers result in most efficient stacking and handling of containers with special equipment. Furthermore, the containerisation leads to cost savings mainly by reducing manpower and, therefore, labour costs. In addition, a steel cover provides a better protection against cargo damages. The global economic crisis with its peak in 2009 had a significant impact on the volume of international container transport as can be seen in Fig. 1. However, it is also apparent that a re-increase has taken place since 2010, but not as large as predicted some years ago (see, e.g., Heymann (2006)). Here, for 2006 to 2015, an annual increase in container throughput in seaport container terminals of 9 % was forecasted. This would result in an increase of 83 % for the years 2008 to 2015, but this has never occured to that extent.

It is difficult to estimate whether and to which extent the container transport will grow in the future (see, e.g., Halim et al. (2017)). The relevant environment is extremely complex and is determined by many influences that cannot be calculated exactly. Usually, companies are moving within the environment of buying, long-term leasing, short-term leasing and repair including the question where to bring in or phase out containers and manage empty container repositioning and usage (see, e.g., Stahlbock and Voß (2010); Varshavets et al. (2013)). These developments are associated with a spatial adjustment of production site locations and manufacturing structures. In this context, e.g., the cost structures in the global division of labor in manufacturing processes can change significantly, in particular with regard to work shares in the low-wage sector. However, a true cost advantage of producing in these areas can be heterogeneously dicussed (see, e.g., Dixit et al. (2019)). Another aspect is the impact of Additive Manufacturing (AM, 3D printing) on global production and logistics structures (see, e.g., Weller et al. (2015); Rayna and Striukova (2016); Sasson and Johnson (2016); Attaran (2017); Ben-Ner and Siemsen (2017); Fratocchi (2017); Jiang et al. (2017)), which can only be roughly estimated so far. For example, some observers estimate that up to 37 % of container shipping operations will be threatened by AM (see, e.g., Strategy& (2015)). Others see AM only in a niche role in logistics and do not expect a huge disruptive effect (see, e.g., Millar (2016)). However, these changes might have a considerable influence in the future. The exact speed and direction of evolvement remains an open issue, but as a result of such developments it is likely that growth in container transport may be impacted to some extent (see, e.g., Halim et al. (2017)). Furthermore, even if this is bound to speculation, there might be a revitalization of "let’s make it at home" due to the Covid-19 pandemic in order to be more independent from countries like, e.g., China and make supply chains more stable and resilient (see, e.g., Keating (2020)). A growing number of businesses are currently rethinking their global manufacturing strategies. These reshoring initiatives would aim at building up own production capacity again in the own country as well as keeping know-how. This reverse globalization would then result in a decreasing world-wide shipping of goods. In order to counteract decreasing freight rates and continued uncertainty in maritime transport, shipping lines form alliances or cooperative agreements (Clott et al. 2018). On the other hand, the container vessel size is continually increasing to meet economies of scale at sea, but creating capacity and operating problems on the port-side (Jeevan and Roso 2019).

The size of the global shipping container fleet (standard containers) is not exactly known. There are no exact records because nobody is responsible for counting containers, but it was estimated in 2013 that about 34 million TEU^1^ existed with approximately 93 % dry containers, 6 % reefers, and 1 % tanks (World Shipping Council 2019). A more elaborated estimate with some explicit and commented calculations is provided by Consultantsea Ltd. (2016), resulting in a total of 43 million shipping containers or approximately 72 million TEU (23 million shipping ’in service’ containers or 38.5 million TEU, 14 million ’ex-service’ shipping containers or 23.3 million TEU, 6 million ’new’ shipping containers or 10 million TEU). It is pointed out that many published estimations only consider ’in service’ containers. That might explain some distortions between all the circulating figures and estimations. In van Leeuwen (2016), the estimated total fleet in 2016 is 40 million TEU, or over 26 million individual units, whereas in Drewry Maritime Advisors (2017) the estimated number of containers is 20 million TEU in 2016. In relative terms, it is said that around 90 % of the world trade (in terms of weight) is carried on seaways either by bulk carriers, tankers for liquids, or container vessels (International Chamber of Shipping 2019).

### Sizes, types and construction of containers

Up to the present, generally accepted principles have been developed with respect to outline dimensions, construction forms, and materials (Strauch 2018): The ISO R-668 fixes the container dimensions to a width of 8 ft, a height of 8 ft (or even 8.5 ft ’standard’ and 9.5 ft ’high cube’), and a length of 20 ft, 40 ft or 45 ft.^2^ The majority of containers complies with those ISO standards. Different construction forms like, e.g., open top, open side, flatrack for out-of-gauge cargo, or tanks for liquids exist. They reflect different interests of forwarders and ship-owners. As it would be beneficial for the ship-owner to transport only standard containers, it is necessary for the forwarder to use means of transport fitted to different types of goods, so that stacking is safer, quicker and, therefore, more economical.

The basic frame, as the weight-bearing element of all standard containers, is made up of steel. Depending on the quality and planned lifespan, it consists of weathered, corrosion-resistant construction steel (COR-TEN) or of SPA-H (’superior atmospheric corrosion resistant steel’). The frame provides the stability and stacking capacity (see, e.g., Containerbasis (2016a)). However, the walls and the roof panel of the container are rarely faced with strains and contribute only to the protection of the goods inside. A material like steel, aluminium or plywood (GRP/glass fibre reinforced plastic coated) is used for the panels, and the container is named after the material. Due to the cost advantage of steel, 85 % of the containers worldwide are made of steel. A plate on the container displays information about the used construction materials, which is important for later repair decisions (Strauch 2018). The container floor is mostly made up of wood. Although the material is rather expensive, it offers the advantage of being more resistant and elastic which enables a cost-effective repair only in subdomains. A disadvantage exists in its open pores, which makes soiling and infestation of parasites occur more easily. Therefore, the wooden surfaces are impregnated or sealed, and the quarantine regulations of the country of destination can dictate certain rules for impregnation (Transport-Informations-Service 2016a).

For safety reasons, the ISO sets minimum requirements to loading capacity and stackability. Stacking of at least six ISO containers of full maximum weight must be possible. In fact, up to 8 or 9 modern containers can be stacked. The approved stackability of each container is declared on its information plate (Strauch 2018).

### Costs

**Fig. 2:**
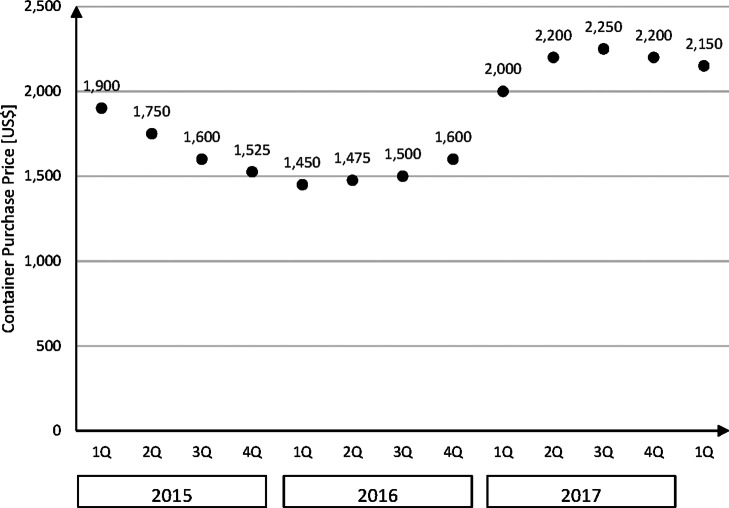
Average price of a new 20 ft container; Data source: Drewry Shipping Consultants (2018b)

The costs of purchasing a container depend on the type and construction form. The price is related to the current price of steel, to supply and demand in the world market, as well as to the individual purchase conditions of each buyer. The price for a steel container was not constant over the years. In 2011, the average price for a 20 ft container was approximately 2,700 USD. In 2015, it was about 1,900 USD, in 2016 about 1,450 USD. In 2017, the price increased to approximately 2,200 USD (Buss Capital GmbH & Co. KG 2017; Drewry Shipping Consultants 2018a). Figure 2 shows the average price for a new 20 ft standard container for quarters from 01/2015 to 01/2018.

**Fig. 3:**
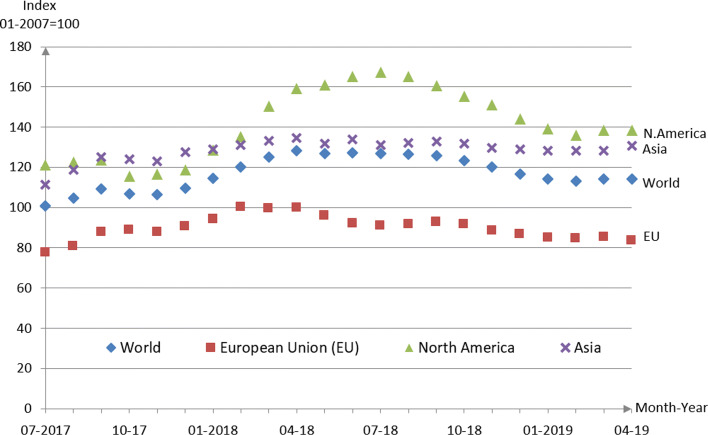
MEPS – Shipping Container Steel Purchasing Price Index, July 2017 – April 2019, January 2007=100; Data source: Management Engineering & Production Services (2019)

In Management Engineering & Production Services (2019), the ’MEPS - Shipping Container Steel Purchasing Price Index’ is shown. The index is based on January 2007=100, with a world index development from May 2016 with 92.2 to, e.g., April 2017 with 104.7, 2018 with a peak of 128.4 in April, falling back to 113.1 in February 2019. Regional developments in the EU, North America and Asia show similar sub-indices. Figure 3 shows data from July 2017 to February 2019.

### Ownership

Leasing companies are accounted for 55 % of container purchases in 2017. The fleet size of shipping companies increased by 2.4 % whereas the leased fleet size increased with 6.7 % so that lessors owned about 52 % in total in 2017. This trend is going to continue. It is estimated that the lessors will own 54 % by 2020 (Drewry Shipping Consultants 2018b). Similar figures are mentioned by Haralambides (2016) (about 50 % are owned and managed by container leasing companies) and (Poo and Yip 2019) (lessors own about 50 to 60 % of the global container fleet). Shipping lines^3^ regulate their container pool based on their current financial situation and their planned growth. Large companies handle a container stock of hundreds of thousands or even several million TEU (see, e.g., Hapag Lloyd AG (2019)).

## Damage of a container

**Fig. 4:**
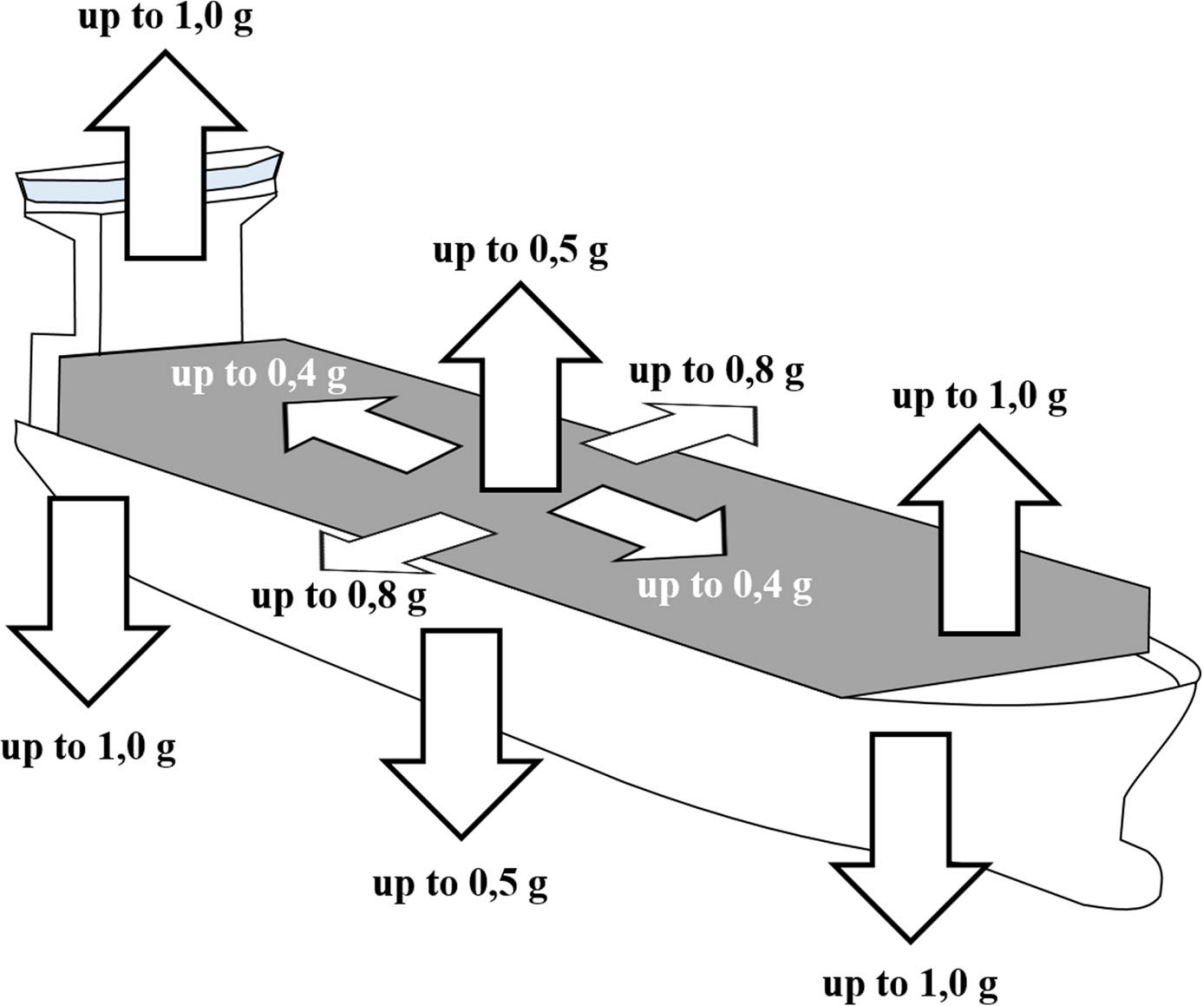
Potential acceleration during maritime transport depending on the stowage place of the container (based on Hapag-Lloyd Container Line – Special Cargo Department (2005), p. 5)

Manufacturing equipment or any other product of a certain grade of quality and price should be or is designed and built to ensure successful operation through the anticipated service life. However, deterioration principally commences as soon as it is commissioned (Muchiri et al. 2014). Like manufacturing equipment, containers can also be damaged for different reasons. One cause is material overstressing in which the container is folded or bent if the material characteristics are exceeded. In Hapag-Lloyd Container Line – Special Cargo Department (2005), it is distinguished between static strain, i. e., weight force in the stack during stowing and stacking, and dynamic strain, i. e., acceleration forces during loading, through ship movements such as rolling, pitching, and yawing during maritime and overland transport or through collisions with containers. Although the cargo inside of the container is mostly safeguarded by sufficient wedging at the horizontal level, the vertical component is entirely neglected (Knott 2000). The vertical force component due to ship movements can reach values up to 2 g and is, therefore, substantially larger than the horizontal force component with values up to 0.8 g (see Fig. 4).

Damage can also be a result of stacking faults because stack weight restrictions are often neglected, especially on chartered vessels. Against regulation, heavy containers are placed on the top of the stack when it is planned to unload those before the lower stacked ones on the route. Due to the costs, the securing system has become downgraded over time with inferior components being used (Knott 2000). This results in the risk that lower containers may shift or become clinched together. Furthermore, damages can be incurred due to varying outside temperatures during maritime transport among different climate zones. Due to condensation (dew point meter), water can aggregate within the container, which can damage goods and promote the formation of rust (UK P&I Club 2018). Finally, damages caused by human error can never be ruled out.

Possible damage patterns are differentiated into scratches, buckles, cracks, breaks, and leaks (Germanischer Lloyd 1978). All classified damages can differ, of course, in location, orientation, and degree and can effect walls, roof panels, floors, cross-rails, and side-rails, as well as corner posts.

There is a lack of publicly accessable damage statistics for containers. According to (Hjortnaes et al. 2017), failure rates for empty containers are high with 20 to 25 % (based on Maersk Line data). In Fransoo and Lee (2013), it is calculated that a container was on average handled about 3.7 times in 2008 (including empty handlings; the authors divided the world container throughput of ports, 506 million TEU, by the worldwide containerized liner trade, 137 million TEU). Handling (picking, moving, delivering) means potential risk for container damages, i. e., the more often a container is moved the higher is the risk for damage of the container. However, some statements to the damage frequency may be derived from cargo claims. If the cargo is damaged, in most cases the container is damaged, too. For containerships, the frequency of all cargo claims increased from 8 % in 2005 to 26 % in 2014 for claims above USD 5,000. However, the frequency for claims below USD 5,000 decreased from 50 % in 2005 to 33 % in 2014. Table 1 provides an overview for frequencies per different loss codes, meaning different fractions, which are more or less related to container damages (The Swedish Club 2016). The main figures above a frequency of 3 % are presented (the shown percentages do not sum up to 100 because only partial data is presented).

**Table 1:** Frequencies per loss code (>3 *%*) – claim categories – claims 5,000 – 3,000,000 (USD), period: 2005–2014; Data source: (The Swedish Club 2016), p. 22

Claim	Percentage [%] of all claims
Improper cargo handling, shore-side	14.50
Flooding of hold	13.36
Heavy weather	12.60
Poor monitoring/maintenance of reefer unit	7.25
Reefer mechanical failure	7.25
Leaking container	6.49
Improper cargo handling, shipside	6.11
Insufficient lashing/securing by shipper	4.58
Collision	3.82
Insufficient lashing/securing by stevedore	3.05

## Repair of a container

When considering a repair, the extent of a damage to the container must be estimated. Large classification societies like ’Germanischer Lloyd’ developed repair recommendations for containers together with shipping companies, leasing societies and repair companies quite early. With these fundamental guidelines or de facto standards for repair, the safe and economic repair of a container should be guaranteed in terms of transport security, dimensional accuracy, weathertightness, and customs seal (Germanischer Lloyd 1978; 1995). As a consequence, rusting, corrosion, buckling, or light scratches will not be classified as repair-requiring damages if the proper use of the container remains guaranteed. Heavier damages to the container, which do not permit the continued proper use of the container, require a mandatory repair, for which detailed instructions are described by Germanischer Lloyd (1978). As an exception, a density check is always performed for tank containers after an executed repair (see, e.g., Germanischer Lloyd (1978), (1995)). There are other societies, companies or services such as ’DNV GL’ providing container certification for ensuring that containers meet requirements for safety, stability, and usability.

### Repair standards

To sustainably grant a certain level of repair quality, it requires the consideration of both economic and security-technical aspects (IICL Technology Committee 2000). To make this individual decision-making process as simple as possible, the following repair standards have been developed committing a trade-off between the lowest repair effort and the highest operational safety:

- IICL: The ‘Institute of International Container Lessors’ (IICL) has developed this standard in cooperation with the ‘International Chamber of Shipping’ (ICS), which is a main association of shipping companies. It is globally used and widely accepted. On the 1st of November 1996, the 5th version of IICL was published and remained in place until the 1st of August 2016 when the updated 6th version with a few changes was released (IICL-ICS 1996; Container Owners Association 2016). This standard is written in tabular form to provide a good overview and explains for every single part of the container how to perform a proper repair.
- UCIRC: Another standard is provided by the ‘Unified Container Inspection & Repair Criteria’ (UCIRC), 3rd edition (April 21, 2004) (see, e.g., International Chamber of Shipping (2004)) which is developed independently by the ICS. It is applied to all standard steel containers. Here, damages of a container are estimated according to their severity and differentiated between acceptable and unacceptable damages. The given tolerances in terms of deformation and buckles are chosen in a way that the container remains operational with the least amount of effort. This standard is also written in tabular form.
- CIC: The ‘Common Interchange Criteria’ (CIC), developed by the ‘Container Owner Association’ (COA), was published in its 1st version on the 11th of March 2011. The CIC-standard tries to combine and harmonize the IICL and the UCIRC standards. Arranged according to assembly and written in tabular form as well, this standard gives an overview of different damages and respective repair recommendations without describing concrete repair procedures. Compared to the guideline in IICL 6, CIC has a higher damage tolerance. Overall, the aim of the harmonization approach was to enable shipping lines to benefit from an elimination of unnecessary repair resulting in reduced repair costs, and to provide a more environmentally friendly approach by reducing container handling. It was announced that starting with the IICL 6, the CIC would follow future updates and revisions adopted on the IICL inspection criteria (see, e.g., Container Owners Association (2016); IICL-ICS (2016)).

#### CSC plate

**Fig. 5:**
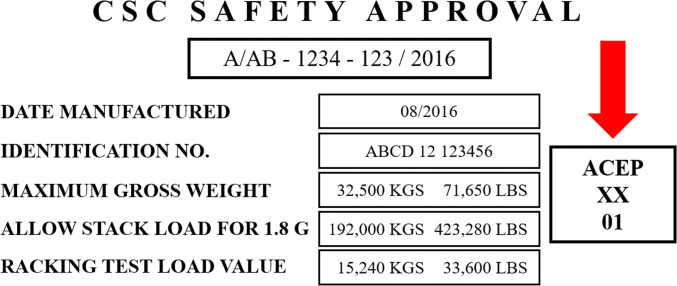
ACEP-note on CSC plate (based on Strauch (2018), Chapter 3.1.2)

In addition to the development of repair standards the ‘Container Safety Convention’ (CSC) has been adopted to obtain a high level of security in terms of human life during the transshipment, stacking and transport of containers. This convention applies to every container which is used for international transport (Strauch 2018). According to this arrangement, containers are checked in terms of their condition and operational safety which is then documented by issuing a CSC plate (Germanischer Lloyd 1995). The CSC plate has a standardized structure (see Fig. 5) and is attached to every container (Germanischer Lloyd 1995). Containers get the plate after their first classification, e.g., when new production series are approved and introduced.

The CSC plate is the only mandatory regulation for containers to provide safety standards for all parties. Therefore, the convention involves returning statutory audits comparable to the MOT test for motor vehicles. Every contracting state is responsible for having effective procedures in terms of examining, visiting, admitting and maintaining the container in a proper way and regulates these issues in its respective federal law.

The plate for a new container is valid for 5 years at first. Afterwards, the plate’s validity can be extended by 30 months each time. A container with a valid CSC plate is supposed to be safe according to the convention (Titan Group 2019).

#### ACEP

Many container owners are members of the ‘Approved Continuous Examination Program’ (ACEP) and, as such, are listed in a database. This program substitutes the fixed returning statutory audits according to the CSC. In this program, the members are obligated to check their containers in regular maintenance procedures independently and to perform repairs as may be necessary (Strauch 2018). The evidence of a participation in this program is noted on the CSC plate (see Fig. 5).

Due to the continuous control of the container, possible damages should be detected and removed earlier than in the usual fixed returning statutory audits (Transport-Informations-Service 2016b). Through this, the quality of the whole container fleet is influenced in a positive way and possible expensive repairs can be avoided in the future.

If a container with an invalid CSC plate or improper application of an ACEP-note is still transported, the container owner will have committed an administrative offence and will incur a fine according to respective federal law.

### Used containers

Used containers can be differentiated into four grades according to their condition. In general, the grade depends on the degree of damages. Table 2 shows the different criteria for each grade.

**Table 2:** Criteria for damage grades; Data source: (Containerbasis 2016b)

Grade	A	B	C	D
Container is	excellent	good	warehousing	inferior
in... quality				
Repaired?	Yes, to	Yes	Yes, with steel	No
	IICL 5 or 6		or aluminium	
Damage?	Small buckles,	Buckles and	Buckles and	Strong
	small scratches,	scratches in	scratches in	damages
	no limitation in	light to middle	middle to strong	
	transport of goods	extent	extent	
Rust-extent?	Light inside or	Light to stronger	Middle to strong	Existing
	outside	inside or	inside and	
		outside	outside	
Wind-weather-	Yes	Yes	Yes	Depends
tightness?				
CSC plate?	Valid	Valid	Invalid	Invalid
Suitability?	Transport of goods,	Export, ware-	Warehousing	With fortune
	warehousing,	housing, freight		warehousing,
	leasing	suitable shape		often used as
				noise barrier

### Repair procedure

If damage to a container is detected in a port, the R&M will not be performed necessarily in this port. First, the harbour needs to have an R&M-facility and finally the container owner decides where and how the damaged container should be repaired. At the beginning of containerization, it was standard practice to repair the container as close as possible to the place where the damage occurred (United Nations – Economic and Social Council – CEPAL – Economic Commission for Latin America 1982). Nowadays, the decision about where and how to repair the container is up to the owner and is more characterized by economic considerations. Thus, the lowest effort is always compared with a possible yield. In this context, a very interesting task for future research could be the formulation of an optimization problem deciding the place and time of repairing a damaged container (taking the model proposed by Hjortnaes et al. (2017) into account, which deals with the repositioning of damaged empty containers in order to reduce pollution, resources, and costs). At this stage, focusing on the following possible cases should be sufficient:

1. Regardless of which port the container was in when the damage was detected, the owner will allow the container to be repaired immediately if a sufficient yield with a direct follow-up business will be generated.
2. If there is no direct follow-up business for the damaged container, the repair decision depends on whether the container can be repaired in a port of low-wage countries instead of an R&M-facility belonging to the container’s owner. However, because all larger shipping companies want to benefit from these low-wage countries, problems with R&M-facilities of these countries occur with respect to capacity or priority of handling different repair requests. Furthermore, some R&M-facilities must entirely reject repair requests as well. For those reasons, large shipping companies have installed, e.g., ‘super depots’ for their containers around the world, where especially specific and difficult repairs are performed. Damaged but transportable containers from other locations are carried to these depots using empty runs, i. e., empty slots on a vessel’s route. After the repair these containers can be used with low profit margins at other locations again.
3. An exception is the ‘emergency repair’. This repair is always performed immediately to ensure safety during the further transport of the container and the arrival of its goods to the final destination. Additionally, a reefer requires special handling because on top of physical and temperature-related damages also electrical damages can occur due to the electrical components responsible for cooling the container. Action must be taken immediately in such cases, because otherwise the goods will be harmed (e.g., nucleation in foods) (Transport-Informations-Service 2016c). For such emergency repairs special mobile repair teams are deployed in order to minimize the possibility of rising costs by exceeding the berthing time of the vessel (see, e.g., United Nations – Economic and Social Council – CEPAL – Economic Commission for Latin America (1982); Destefano (1983); Pacino (2013)).

After arriving at an R&M-facility, the container first undergoes an inspection. There are prepared checklists for this purpose in every facility. The container is examined completely, and all damages are registered on the checklists. Special attention is paid to the floor of a container due to contamination, splintered wood or protruding nails, all of which can harm future cargo. After that inspection, the R&M-facility estimates the costs for the repair, based on check tables and repair standards. If the estimate is accepted, the container owner or local agents will check (in about 60 % of all cases) whether all damages listed in the cost forecast really exist and must be repaired necessarily according to a certain repair standard. The client is kept informed by daily status reports during the repair process (Destefano 1983). Furthermore, an additional test can be recommended in some cases, e.g., after extensive repairs, before the repaired container is returned to the owner. There, safety factors compared with different failure types, e.g., material fatigue, are checked. The safety factors constitute minimum requirements for materials (Germanischer Lloyd 1995).

In general, responsibility and payment in the case of damage are directly connected, whereby it can sometimes be very difficult to definitively establish responsibility in a long logistic chain. Therefore, it is first decided following the exclusion principle which part of the logistic chain can be released from responsibility. Thus, it is narrowed down to who is (most likely) responsible (Strauch 2018). To determine responsibility, the ’Equipment Interchange Receipt’ (EIR) is significant. This receipt shows the date, owner, container number, type, color, and the size of a container (usually written in English). Regarding reasons for responsibility, a visual test of the container for damages is performed at every interchange within the logistic chain. Both, deliverer and recipient sign the EIR which confirms in-/out-gate (goods in/goods out) of the container (CMA CGM Group 2018). If a damage that is not already listed is detected during interchange, the repair cost for this damage must usually be paid by the one who has had the responsibility of the container before. It is assumed that the damage has happened during that time. However, sometimes the definite identification of the origin of the damage is possible due to certain circumstances, e.g., if goods inside of a container have not been loaded and secured in a proper way and the damage has occurred during transport. Naturally, there is the opportunity to insure the container against damages. With respect to container damages there are two commonly used insurance types to be distinguished. The differentiation depends on the viewpoint as to whether a container is merely packing material for goods (cargo insurance) or a good in itself and represents tangible value to the owner (container hull insurance) (Strauch 2018).

## Damage prevention

From the analysis of container damages, different ways of damage prevention can be deduced. The essential advantages of damage prevention are the smooth transport of the goods without complaints, the avoidance of high repair costs, and the reduction of the potential danger to people, goods, and vessels.

The container side is an area with a huge damage potential which can be reduced preventively by always checking a container using a checklist before entering or leaving the container depot. A special focus should be given to containers with apparent repair signs. Container owners using the following checklist are usually also members of the ACEP-program (Hapag-Lloyd Container Line – Special Cargo Department 2005, pp 15):

- External checklist:
  - No holes or cracks in walls and roof.
  - Doors can be operated easily.
  - Proper function of locking devices and handles.
  - No adhesive labels from previous cargo, e.g., IMDG (‘International Maritime Code of Dangerous Goods’) placards. Dangerous goods labels are only allowed if dangerous goods are inside.
  - Flatracks: stanchions (if ordered) should be complete and inserted properly.
  - Open top: roof bows should be complete and inserted properly.
  - Open top: tarpaulins and ends are undamaged, correct size.
  - Hard top: the roof is undamaged; roof fastening fits properly and is accessible.
- Internal checklist:
  - Container is waterproof. Test method: stand inside the container, close all doors tightly and examine for any light coming through cracks, holes, door gasket, etc.
  - Container is completely dry inside. Wipe out all condensation or white frost to avoid corrosion and moisture damage to the cargo.
  - Container is free of dirt and cargo residue, clean and odourless.
  - Floor: no nails or other protruding objects.

A suitable container and proven equipment should be selected for transport to provide an optimal level of security for the goods (Knott 2000). The guidelines of the CTU-guide (‘Cargo Transport Unit’) should be followed as well. However, the best developed stowing and stacking rules will not pay off in practice, if the rules wind up being used in the wrong way by a human being. Therefore, the professional training of staff is very important. This can involve, e.g., training programmes, workshops, or internet-based training videos (IICL-ICS 2016), as well as Augmented Reality devices.

The container on a vessel is affected by vertical and horizontal forces during transport. The vertical force can be absorbed by using cell guides (vertical guide rails) so that a container is only affected by the horizontal force. If a container cracks under the stack weight, the magnitude of damage will be limited, because the upper and surrounding containers are stabilized by the guide bars (Strauch 2018). In order to counteract these forces at sea optimally, containers should always be stacked lengthwise so that the container’s stronger side walls can absorb the greater side forces. According to ISO 1496/1 the side walls of a container are to absorb up to 60 % of the maximum payload in comparison to the front-end wall with only 40 % (Hapag-Lloyd Container Line – Special Cargo Department 2005). If it is still necessary to stack in a crosswise direction, this must be considered in terms of securing the cargo and packing of the container (Strauch 2018). In addition to that, the doors of the container should not be orientated to the bow of the vessel because then sea foam could break into the container more easily.

The shipmaster can also counteract possible risks for container damages by altering the vessel’s operation, and by choosing the right route (i. e., bypassing bad weather conditions with severe winds and high waves). However, sticking to the vessel schedule design is very important (Pasha et al. 2020), because delays to scheduled arrivals can result in longer waiting times before ports (Clott et al. 2018) and finally to violated delivery times of the loaded cargo. Thus, successfull shipping companies arrive in ports on time more often than not (Lang and Veenstra 2010).

After arriving in a terminal, containers are frequently handled in various ship and hinterland operations by different carriers like gantry cranes, yard trucks, or reach stackers. In order to cope with larger vessel sizes and rising container numbers, different improvements in handling equipment were made like transporters with multiloading capabilities or algorithm-based storage systems for, e.g., minimizing rehandles (Kim et al. 2012). Due to limited areas, container handling equipment practically operates in close proximity. Thus, effort is made in developing and implementing different collision prevention technology using, e.g., laser scanners, radar sensors, wind scanners, or rear-drive assistants on cranes, stackers, trucks, or transporters (Port Equipment Manufacturers Association (PEMA) et al. 2012). Besides from collision prevention, the use of electronic data processing (EDP) assisted systems can help with calculating optimal bay plans, reducing rehandles, and detecting incorrectly declared container weights during loading processes (see, e.g., (Wika Mobile Control 2018)). In particular for poorer countries, a general improvement in the port infrastructure leads to better logistics performance and reliability with increasing seaborne trade and economic growth (Munim and Schramm 2018). To the best of our knowledge, automated gantry cranes with sensors measuring outline dimensions, speed, and distances while positioning the container for preventing collisions and damage of containers are not widely implemented within the terminals so far, since they are not yet well enough evolved. In addition, the International Maritime Organization (IMO) introduced specific SOLAS regulations ((International Convention for the) Safety of Life at Sea) regarding the weight of containers. According to Chapter 6 in this regulation, it is legally binding since July 1, 2016, to verify the gross mass of containers (resulting in a Verified Gross Mass (VGM)) before loading containers onto a vessel. The shipper himself is the responsible party which has to determine the VGM. The determined VGM has to be approved by an authorized person, by (digitally-)signing the weight. If the shipper is not under the ownership of well-calibrated approved weighing equipment meeting national standards, the VGM can also be determined by a third-party charged by the shipper. The process and responsibilities can be tracked by the documents and signatures. Without a VGM, the container is not loaded onto a vessel (International Maritime Organization 2014; 2016). These regulations aim at preventing, e.g., collapses of stacks and the loss of containers overboard (International Maritime Organization 2019). So far, no evaluation or statistics on the effect of the IMO/SOLAS regulations have been found. The research gap is reflected in a current call for participation in a survey and evaluation of the VGM regulations of the University of Liverpool (European Shippers’ Council 2019).

## Decision on repair

Previously, it was shown that R&M of a container should be examined in a sophisticated way due to the different influencing factors and regulations, since a variety of possible damages, diverse container properties, uncertain and rapid changing overall business and order situations, altering steel prices, and a possible secondary market exist and result in various repair considerations and decision opportunities. With this background, the following decision model can be used independently from the owner and the number of owned containers as well as other certain business circumstances. The model should, for each single container, provide guidance as to whether a repair should or should not be performed considering different actual parameters.

A first approach can be based on cost accounting methods or investment appraisals, i. e., focusing on costs (and returns) and considering a repair decision as an investment decision. The investment appraisal provides a static and dynamic method. The dynamic method considers the temporal sequence of an investment and takes rates of interest into account in contrast to the static method. Hence, a decision model can be developed based on a dynamic investment appraisal (discounting method). A payback period approach, which contrasts the possible earnings from further use with the expenses of an investment, can be reasonable as well. If a payback period approach is used according to a choice between different investment options, the decision with the shortest payback period will be usually chosen. Another calculation of profitability is to consider the capital recovery factor taking changes according to the structure of costs and expenses in different observation years into account. All costs and expenses accumulating during the decision process are gathered in a static way.

**Fig. 6:**
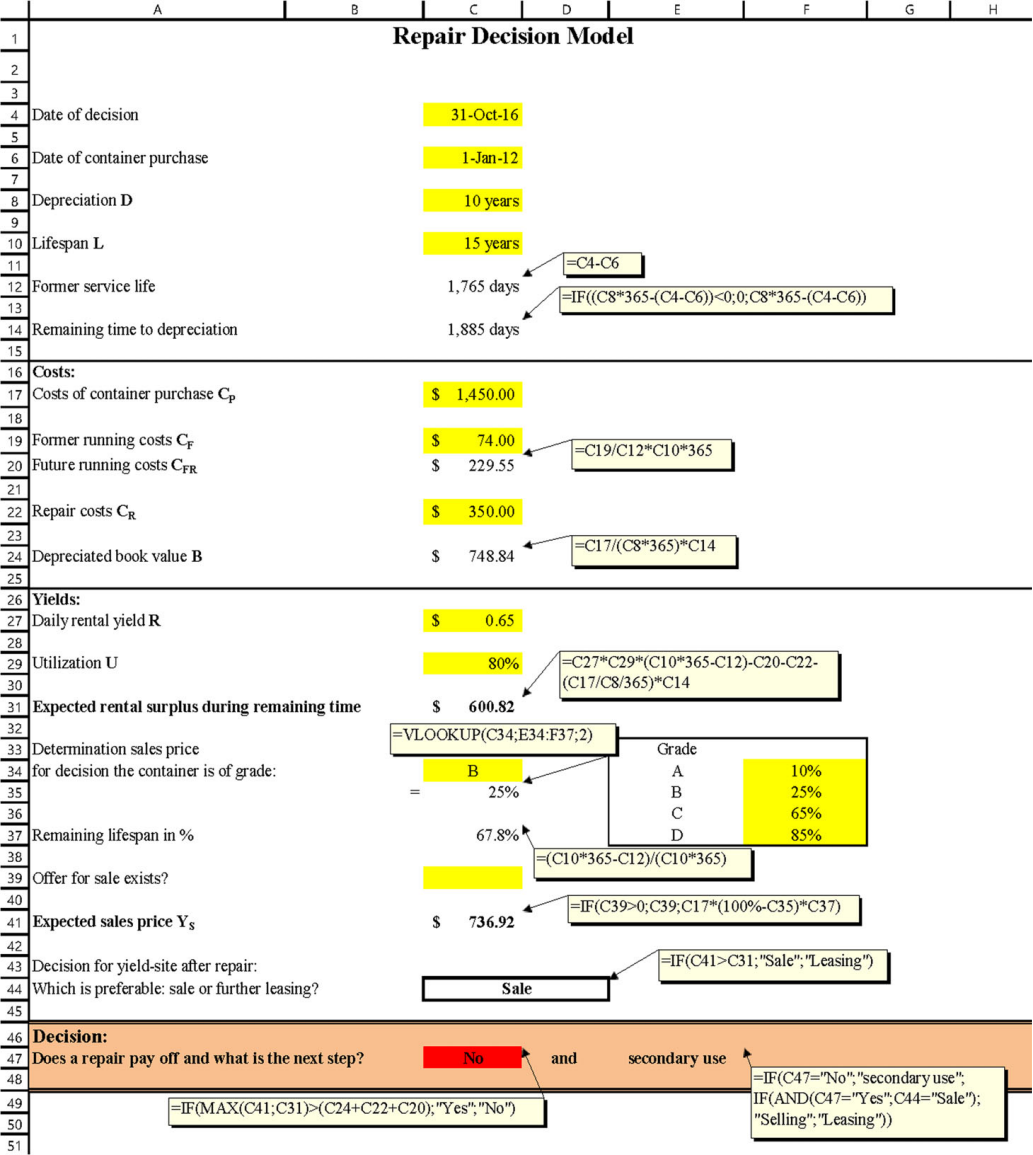
The decision model implemented in Excel with displayed comments; here, case c (see p. 17) is displayed as an example

The developed decision model is subsumed mostly by these profitability calculations mentioned above, whereas none of these methods alone is appropriate. The decision model is implemented as an easy-to-use Microsoft Excel spreadsheet and automatically provides a colour-coded guidance. The model is presented in Fig. 6.

Here, the yellow-coloured Excel cells are input fields for data. The required data can be entered manually by the decision maker. Alternatively, these data can be provided by computer-assisted databases, which identify containers with the help of, e.g., radio frequency identification (RFID) technology and automatically assign information like damage reports, repair cost estimations, customer booking status, target position, usage history, and predicted future, as well as container master data to the respective container (see, e.g., Ngai et al. (2007)). Real data as well as assumptions can be entered in the model and the respective guidance becomes automatically apparent. Finally, it is admitted that not all relevant parameters can be considered in the presented model in order to keep the complexity on a managable level. This means, e.g., that the transport costs to a repair station are not considered, since the container vessel independently takes this route and further stowage issues like the Multi-Port-Master Bay Plan Problem (Wilson and Roach 2000) should have been included, leading to complex solving approaches with multiple side conditions like Mixed-Integer-Programming (Ambrosino et al. 2015) or Large-Neighborhood-Search (LNS) (Pacino 2013). Consequently, the respective guidance depends on the following parameters:

- Date of decision
- Date of container purchase
- Depreciation (*D*): The depreciation of a container is assumed to be linear and contains, e.g., 10 years according to German tax legislation.
- Lifespan (*L*): The average lifespan of a sea container is in the range of 12 to 15 years according to the literature (Containerbasis 2016a; Poo and Yip 2019) or even longer (Haralambides 2016). However, the value can be chosen and entered in the model without any restriction due to different personal experience values. Special container types like, e.g., ‘Flatrack’ or ‘Open Top’ usually exceed a lifespan of 15 years, whereas break bulk cargo containers seldom last as long. Reefers are usually replaced promptly at the end of their 10-year lifespan because technical defects of the cooling system are increasingly impending at that point and because the used technology often becomes obsolete.
- Former service life (days): It is calculcated as the difference between the decision date and the date of container purchase.
- Remaining time to depreciation (days; minimum: 0): It is calculated as the difference of depreciation time and the former service life.
- Costs of container purchase (*C*_*P*_): In this model, the costs of container purchases are gathered under only one field so it is possible for the decision maker to also subsume other costs occurring with the purchase like, e.g., classification costs of a container series, overpass costs or customs duties etc. under the position *C*_*P*_. Only the costs of purchase for a 20-feet standard container are entered in the example calculation.
- Former running costs (*C*_*F*_): In the former running costs, all costs are subsumed which have occurred up to the date of a repair decision, e.g., inspection and maintenance costs or performed repairs. Usually, *C*_*F*_ are quite low in the first years. However, appropriate data was not found in the literature, so *C*_*F*_ is calculated based on assuming costs of 35,000 EUR/p.a. for a person performing work on containers including material. Deducting 15 % for illness and holidays, about 1,730 working hours are available for one employee each year. Given the assumption of an annual maintenance lasting 45 minutes per container, 2,306 containers can be maintained per year and employee. This results in the calculated work performance of nine containers per workday. Entering the former running costs in the model field, the total costs of 35,000 EUR are divided by the number of containers referred to during the operating time. In the example calculation, the operating time is 58 months, so it results in costs of 74 EUR per container.
- Future running costs (*C*_*FR*_): This cost centre involves future *C*_*F*_ until the end of the period under consideration. These costs are calculated based on lifespan and former running costs. If the decision maker has other data he can enter the value in this field.
- Repair costs (*C*_*R*_): Under this position all costs are subsumed which are related to the respective repair procedure, e.g., transport of an empty container to and from the R&M-facility. Usually, a complete cost forecast is provided in these cases.
- Depreciated book value (*B*): The depreciated book value is calculated by the cost of the container purchase minus former amortization expense.
- Yields (*Y*): The yields are determined by either the daily rental yield *R* per day for leasing companies or a rental equivalent savings amount for a ship-owner having a container in his own stock, as he does not need to pay rent for the container. The total yields are then calculated for the remaining time of the container. The figures in literature vary heavily depending on the container type. Furthermore, they are affected by supply and demand, the relative number of pieces, and possibly other factors. During the last decade, the yield fluctuated in the range of approximately 0.38 USD (in 2016) to 0.94 (in 2011) (see, e.g., Buss Capital GmbH & Co. KG (2017); Drewry Shipping Consultants (2018a); Fonds professionell online (2018)). In the calculation example, a value of 0.65 USD was entered (data taken from (Buss Capital GmbH & Co. KG 2006)).
- Utilization (*U*): Here, the hypothetical future utilization for the container is estimated based on the personal experience values of the decision maker according to the general economic and order situation. The value of 95 % used in the calculation example is quoted from (Buss Capital GmbH & Co. KG 2006). According to (Buss Capital GmbH & Co. KG 2017) and (Fonds professionell online 2018), the utilization rates in 2016 and 2017 were about 92 % and 96 %.
- Sales price (*Y*_*S*_): The expected sales price *Y*_*S*_ is either an existing offer of purchase or – if not entered – a value calculated by the model. The value of used containers depends on grades and the price is adjusted further due to supply and demand on the market. Depending on each grade (see Table 2), the costs of purchase can be discounted. The decision maker can freely allocate each discount percentage for every grade in the model. The following values are assumed for the calculating model: The respective loss in proportion to the cost of a container purchase of the grade A is 10 %, the grade B is 25 %, the grade C is 65 % and the grade D is 85 %.

An investment decision regarding the execution of a repair to a damaged container passes a decision process which is divided into three steps: First, the real repair scope must be determined, and in this context these are the repair costs. If further data is procured, the most favourable guidance will be chosen. Following this principle, the decision model can be used to differentiate between the following three basic cases:

- The container will always be repaired and further used/rented if – on account of the entered data – the expected yields in the remaining lifespan are larger than the projected costs.
- The container will be repaired and sold promptly if the yields of selling the repaired container are greater than the projected costs. For this, the model performs automatically a comparison of the expected sales price and the possible yield of further use. In this case, it is necessary to enter a specific offer of purchase. The model puts this value in the field ‘Expected sales price’ and considers that value.
- The container should not be repaired anymore if the possible yields are not high enough to cover the projected costs. In such a case, the economic efficiency can be reached by an appreciation in secondary use. This also considers sustainable aspects since used containers are not generally scrapped if they cannot fulfil their first intended use as well as manufacturing from scratch usually has the highest consumption of natural and fossil ressources. Practically, added values in secondary use are performed by, e.g., conversion in residential, sanitary or construction containers. Then, prices like 6,500 EUR for office containers, 8,500 EUR for sanitary containers and, depending on expansion stage, more than 10,000 EUR for residential containers are common on the market (SBS Containerservice GmbH 2019). More and more R&M-facilities located in Europe have specialized in the secondary market because repair orders have shifted mostly to the Far East due to cost advantages (see, e.g., Großkurth et al. (2011)).
  The presented model does not consider conversion costs explicitly in an input field, because it is assumed that the container owner previously sells the damaged container. Thus, the conversion costs can be attributed to the container purchaser.

## Conclusion and outlook

Despite intensive improvements and the application of EDP-assisted technology and sensors, damages to containers occur everywhere in global trading and the transport of goods in sea containers nonetheless. Even if a repair decision is affected by certain circumstances, such as the utilization of the container fleet, the general financial situation of the company, and steady competitive constraints, the container owner must always decide on every single case. The proposed decision model can support the decision maker by giving a first guidance on performing R&M with the consideration of various critical parameters, leading to an economically feasible repair decision with a sustainable inclination.

However, the model is for sure not able to reflect all circumstances effecting a decision. The model should actually be based on a dynamic method which discounts the future sales price of the container as well as further yields from usage in view of the remaining lifespan to the net present value. However, this could be disregarded due to the current central bank rate of approximately 0,0 % in Europe. In addition, it would be necessary to consider possible future damages during the remaining lifespan in order to define the remaining running costs in a more precise way. Extensive damage statistics would be very helpful here, but unfortunately such information is not gathered. The proposed model further simplifies by assuming that a repaired container is as good as a new container (grade A) and that no other repairs will likely occur during the remaining lifespan. Further development of the current model will be necessary if the container owner has data on the future damage development of his containers.

It should be noted that decision-making with respect to R&M of sea containers can be affected significantly by events like, e.g., existing political economic embargoes to certain countries, current changes in the business and tax law, changes in precepts and agreements between certain countries, as well as unpredicted and sudden severe weather conditions and natural catastrophes. These kinds of influences are not reflected in the presented decision model. Further research should aim for developing an even more elaborate decision model by combining repair decisions with empty container repositioning. Different R&M-facilities should be reflected in the model in a way that the place where a container should be repaired is also part of the calculus for the repair decision.

The combination with data-driven machine learning strategies and tools for, e.g., solving pattern recognition and forecasting tasks also involves a potential for the proposed model for decision support and business intelligence. Modern concepts of data-driven analytics (predictive: ‘What will happen?’, prescriptive: ‘What should we do?’, and adaptive: ‘How should the system adapt to the latest changes?’) might be fruitful for improving the logistics processes and decisions in the field of R&M (see, e.g., Heilig et al. (2019)). Thus, best practise approaches could be derived from a sensitivity analysis that are generally applicable for entire container fleets with applied standard maintenance procedures.

## Data Availability

Not applicable.

## Abbreviations

ACEP: Approved Continuous Examination Program
ADS: Allgemeine Deutsche Seeversicherungsbedingungen
AM: Additive Manufacturing
CIC: Common Interchange Criteria
COA: Container Owner Association
COR-TEN: Specific type of steel (with corrosion resistance and tensile strength)
CSC: Container Safety Convention
CTU: Cargo Transport Unit
DNV GL: Accredited registrar and classification society, merger of Det Norske Veritas (Norway) and Germanischer Lloyd (Germany)
EDP: Electronic Data Processing
EIR: Equipment Interchange Receipt
EU: European Union
EUR: Euro
GRP: Glass Fiber Reinforced Plastic
ICS: International Chamber of Shipping
IICL: Institute of International Container Lessors
IMDG: International Maritime Code of Dangerous Goods
IMO: International Maritime Organization
ISO: International Organization for Standardization
MOT: Ministry of Transport
R&M: Repair and Maintenance
SOLAS: Safety of Life at Sea
SPA-H: Specific steel grade (superior atmospheric corrosion resistant steel)
TEU: Twenty Feet Equivalent Unit
UCIRC: Unified Container Inspection &Repair Criteria
USD: United States Dollar
VGM: Verified Gross Mass

## Footnotes

1 According to their length, containers are called 20-foot standard container (TEU = Twenty Foot Equivalent Unit) or 40-foot standard container (FEU = Forty Foot Equivalent Unit).

2 The length of a 20ft container is in fact 19 ft and 10.5 inches. Furthermore, 10ft and 30ft containers are mentioned in the ISO R-668.

3 Shipping lines are companies that physically own and run the container vessels. Some shipping lines own all their containers, some own only a part and rent the rest from a leasing company, and some own none and lease all their shipping containers.
